# Nanohybrids of Silver Particles Immobilized on Silicate Platelet for Infected Wound Healing

**DOI:** 10.1371/journal.pone.0038360

**Published:** 2012-06-05

**Authors:** Chia-Yu Chu, Fu-Chuo Peng, Ying-Fang Chiu, Hsing-Chuan Lee, Chien-Wen Chen, Jiun-Chiou Wei, Jiang-Jen Lin

**Affiliations:** 1 Department of Dermatology, National Taiwan University Hospital and National Taiwan University College of Medicine, Taipei, Taiwan; 2 Institute of Toxicology, College of Medicine, National Taiwan University, Taipei, Taiwan; 3 Institute of Polymer Sciences and Engineering, National Taiwan University, Taipei, Taiwan; University Hospital Hamburg-Eppendorf, Germany

## Abstract

Silver nanoparticles supported on nanoscale silicate platelets (AgNP/NSP) possess interesting properties, including a large surface area and high biocide effectiveness. The nanohybrid of AgNP/NSP at a weight ratio 7/93 contains 5-nm Ag particles supported on the surface of platelets with dimensions of approximately 80×80×1 nm^3^. The nanohybrid expresses a trend of lower cytotoxicity at the concentration of 8.75 ppm Ag and low genotoxicity. Compared with conventional silver ions and the organically dispersed AgNPs, the nanohybrid promotes wound healing. We investigated overall wound healing by using acute burn and excision wound healing models. Tests on both infected wound models of mice were compared among the AgNP/NSP, polymer-dispersed AgNPs, the commercially available Aquacel, and silver sulfadiazine. The AgNP/NSP nanohybrid was superior for wound appearance, but had similar wound healing rates, vascular endothelial growth factor (VEGF)-A levels and transforming growth factor (TGF)-β1 expressions to Aquacel and silver sulfadiazine.

## Introduction

Currently, silver-ion compounds, such as silver sulfadiazine (SS) cream, are applied to burn wounds to promote healing because of their antibacterial and pro-healing effects. [1], [2] Anti-bacterial activity is an important factor for wound healing. Re-epithelization occurs during wound healing and involves the proliferation of keratinocytes and the differentiation of fibroblasts. [3] Keratinocyte proliferation generates an epidermal-skin layer, and the fibroblasts create the contractile force during wound healing. [4] Recent developments in nanotechnology for synthesizing nanometer-size materials may provide an opportunity for enabling effective wound healing due to material surface interaction with cells and tissue. [5] In general, silver compounds are utilized as therapeutic modalities for wound management due to their biocidal activities toward a wide spectrum of microorganisms and their anti-inflammatory effects. [6]–[10] By contrast, the silver-ion compounds may have cytotoxic effects and impair the wound healing process. [11] Furthermore, the material stability and efficacy for wound healing could be tailored by nanotechnology such as controlling the size of the silver nanoparticles.

The properties of nanomaterials that are 1–100 nm in size can be manipulated [12], [13] to affect their functions when interacting with biomaterials and biomedicines. [14], [15] Among the various types of nanomaterials, the clay minerals are ubiquitous in soils and considered to be safe materials for utilization in medical applications. Specifically, montmorillonite clay comprised of primary stack units of multiple aluminosilicate sheets with average dimension of approximately 80 nm×80 nm×1 nm were modified into high?surface?area silicate platelets. [16], [17] Individual platelets possessing high surface charges and surface area are suitable for supporting inorganic nanoparticles such as silver nanoparticles (AgNPs). The properties of the resultant material regarding its size compatibility, geometricshape, and complementary and synthetic feasibility are important considerations when developing a new nanomaterial. Previously, we have found that nanohybrids of AgNPs tethered to silicate clays with a multilayered stack primary structure could be effective in inhibiting a broad spectrum of bacteria. [18] However, the nanohybrids have a general inherent tendency for aggregation into bulk and reduction of the efficacy. The syntheses of new species of nanometer-scaled inorganic materials without aggregation remain a challenge. [19] Our earlier development of the exfoliation technique for preparing nanoscale silicate platelets (NSP) [16], [17], [20] could provide a new material with a high surface area to interact with AgNPs. The Ag immobilized on silicate platelets (AgNP/NSP) was found to possess interesting properties, such as a high surface area and biocidal activity. [21] The goal of this study was to examine the performance of the nanohybrid in the wound healingprocess. *In vitro* assays were performed, including cytotoxicity and genotoxicity tests, to confirm the safety profile and to determine the adequate concentration. Then, mice were used as an *in vivo* model to examine the efficacy of AgNP/NSPs in a mouse skin wound healing tests. The potential of AgNP/NSPs to regulate the levels of cytokines related to angiogenesis, fibrosis, and wound inflammation were also evaluated.

## Results and Discussion

### Comparison of the Cytotoxicity and Genotoxicity of AgNP/NSP, Poly-Ag, and Commercial Silver-ion Drugs

**Figure 1:**
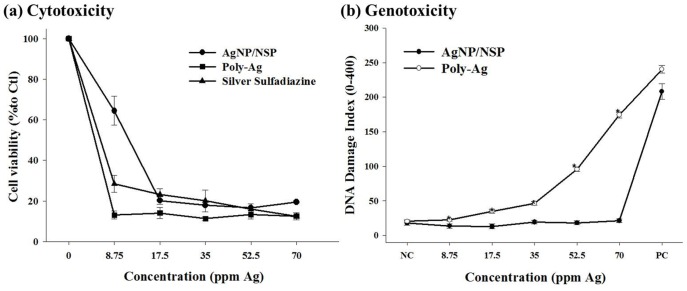
Cytotoxicity toward the human foreskin fibroblast (Hs68) cell line after 12 h of incubation with AgNP/NSP, polymer dispersed AgNP (Poly-Ag) and silver sulfadiazine (SS) using the MTT assay (a). The values are expressed as the mean ± SD from 4 independent experiments. DNA damage in Chinese hamster ovary (CHO) cells by the comet assay showed the average lengths of the cell tails after incubation with different concentrations of AgNP/NSP and Poly-Ag (b). Data are shown as the mean ± SD (at least 200 cell tails were counted in each sample). *P<0.05 at each concentration, Student’s *t* test.

Compared with the six layers of stacked clay in our previous report, single-layered NSPs are fully exfoliated and their ionic charges are exposed on the surface. These characteristics enable the formation of smaller AgNPs (7 nm vs. 26 nm diameters) and provide a larger cell-material contact surface, possibly accounting for the improved antibacterial effectiveness of this new nanohybrid [21]. The cytotoxicity of AgNP/NSPs to human foreskin fibroblast (Hs68) cells was determined with the MTT assay [20], indicating a dose-dependent cytotoxicity. Compared with commercial drugs such as SS and polymer-dispersed AgNP (Poly-Ag), the AgNP/NSPs were found to show a trend of lower toxicity at a concentration of 8.75 ppm Ag. (Fig. 1a).

The role of NSP as a support for AgNP in comparison with organic dispersants was further evaluated with respect to its influence on genotoxicity. The comet assay [22] examines DNA damage by revealing a “comet tail” of DNA fragments with different lengths following electrophoresis. In Fig. 1b, the DNA damage index of each group was calculated by counting the comet tails in Chinese hamster ovary (CHO) cells treated with Ag/NSP and Poly-Ag with silver concentrations of 8.75, 17.5, 35, 52.5, and 70 ppm. [20] By comparison, significantly more comet tails caused by DNA damage were observed in the poly-Ag group than the AgNP/NSP group where mainly spherical shapes of intact DNA were found (*P*<0.05 at each concentration, Student’s *t* test). According to the DNA damage index (the ratio of tails to total comet bodies), no DNA damage could be detected in cells treated with AgNP/NSP at concentrations lower than 70 ppm. In contrast, in the Poly-Ag group, extensive and dose-dependent damage was observed. The control experiments demonstrated no mutagenic effects in 3 different genotoxicity tests for NSP itself [20]. It appears that the platelet-shaped NSPs may immobilize AgNP and impede the nanoparticle penetration into mammalian cells and bacteria. The use of the NSP support may help avoid deposition of detrimental silver in the body.

### AgNP/NSPs Promote Healing of Infected Wound in Acute Burn and Excision Models

**Figure 2:**
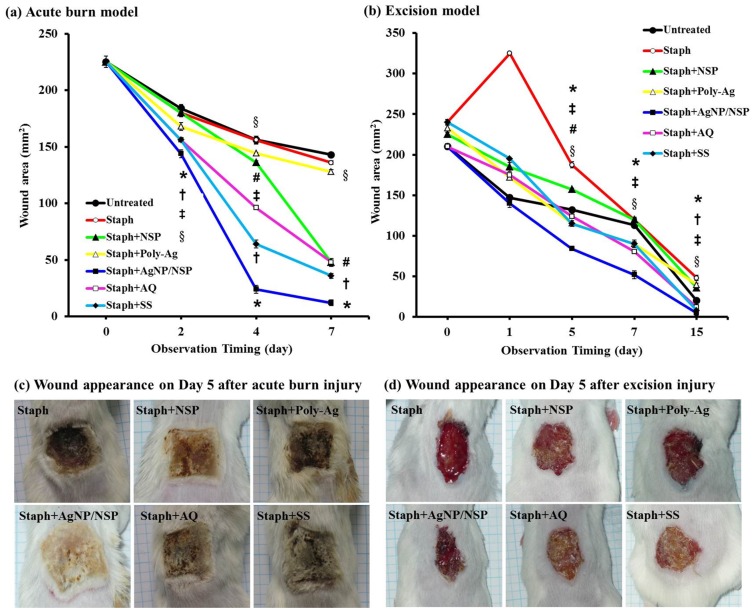
Wound healing rates on (a) acute burn model (b) excision wound model and the photographs of wound appearance on day 5 after (c) acute burn injury and (d) excision injury. The rate of infected wound healing was compared in animals treated with staphylococcus only (Staph), staphylococcus and NSP (Staph+NSP), staphylococcus and Poly-Ag (Staph+Poly-Ag), staphylococcus and AgNP/NSP (Staph+AgNP/NSP), staphylococcus and Aquacel® (Staph+AQ), staphylococcus and silver sulfadiazine (Staph+SS), and control (untreated). The results are expressed as the mean ± SD from three independent experiments in each group. *P<0.05, comparison between AgNP/NSP and the other 6 groups at each time point; † *P*<0.05, comparison between SS treatment with the untreated, Staph, Staph+NSP, and Staph+Poly-Ag groups; ‡ *P*<0.05, comparison between AQ treatment with the untreated, Staph, Staph+NSP, and Staph+Poly-Ag groups; # *P*<0.05, comparison between NSP treatment with untreated, Staph and Staph+Poly-Ag groups; § *P*<0.05, comparison between Poly-Ag treatment with untreated and Staph groups. All the comparisons were confirmed by Student’s *t* test.

Wound healing is a complex process involving inflammation, formation of granulation tissue, re-epithelization, as well as matrix formation and remodeling. [23] Infection may further impede the healing process and result in a non-healing wound. Silver compounds are generally effective for wound healing. [6], [7] The use of AgNP/NSPs could facilitate the healing process owing to their antimicrobial properties. The wound injury test in acute burn and excision models was performed with *S. aureus* as the infective pathogen. For the purpose of comparison, we used a silver concentration of 140 ppm for both Poly-Ag and AgNP/NSPs in the experiments. The concentration is similar to that of the commercially available Aquacel® (AQ). During the healing process, wound contraction or reduction of the open wound area was observed, and the results are shown in Figure 2. In the acute burn model, the wound area in the Staph+AgNP/NSP group was significantly more reduced than in any of the other 6 groups and had significantly smaller wound areas at days 2, 4 and 7 (*P*<0.05, Student’s *t* test, Fig. 2a). AQ or SS treatment also resulted in significantly smaller wound areas compared with the untreated, Staph, Staph+NSP, and Staph+Poly-Ag groups at most of the above time points. However, on day 7 AQ did not show a smaller area than the Staph+NSP treatment; NSP treatment only showed significantly better wound healing than the Staph and Staph+Poly-Ag treatments on days 4 and 7 (*P*<0.05, Student’s *t* test). Smaller wound areas were observed in the Poly-Ag group than in the untreated and Staph groups at days 4 and 7. The Staph+AgNP/NSP group showed the best therapeutic effect, revealing a clean wound surface, improved appearance, and less eschar formation compared with the other silver compounds tested, including AQ and SS (Fig. 2c).

In the excision wound model, a piece of full-thickness skin (1.5×1.5 cm^2^) was excised with sterilized scissors. The bacteria-implanted Staph group showed a persistent discharge from the wound base and delay in wound closure. The treatment with AgNP/NSP significantly improved the wound closure, with a wound area (5±3.5 mm^2^) less than that in any of the other 6 groups, as indicated in Fig. 2b (*P*<0.05 at days 5, 7, and 15). Treatment with AQ also resulted in significantly smaller wound areas compared with the untreated, Staph, Staph+NSP and Staph+Poly-Ag groups at days 5, 7 and 15 (*P*<0.05, Student’s *t* test). SS treatment only reached a significant difference compared with the above 4 groups at day 15. No difference between Staph+AgNP/NSP, Staph+AQ, and Staph+SS with respect to wound closure was observed at day 15.

### The Effects of AgNP/NSP Treatment on the Cytokine Levels

During the wound healing process, many factors contribute to wound closure. This process is executed and regulated by an equally complex signaling network involving numerous growth factors. [23] In this study, we chose VEGF-A, TGF-β1, and IL-6 to represent 3 different aspects of the wound healing process, i.e., angiogenesis, fibrogenesis, and inflammation.

In the acute burn model, the serum VEGF-A levels were found to be significantly higher in the Staph+AgNP/NSP group than the Staph+NSP, Staph+Poly-Ag, and Staph groups (Table 1, *P*<0.01). The VEGF-A levels, however, were similar in the Staph+AgNP/NSP, Staph+AQ, and Staph+SS groups. VEGF-A is an important growth factor involved in vasculogenesis and angiogenesis. It was found that AgNP/NSP had the same effect as the commercial products (AQ and SS) on VEGF levels. TGF-β1 is a protein that controls the proliferation of keratinocytes at the wound edge and increases cell number to promote wound covering through re-epithelization. The results indicated that the serum levels of TGF-β1 were significantly higher in the Staph+AgNP/NSP group than the Staph group (*P*<0.01). The TGF-β1 concentration in the Staph+AgNP/NSP group was similar to that in animals treated with the commercial products, AQ and SS. IL-6 is a pro-inflammatory cytokine that is up-regulated in the early inflammatory phase. [24] The expression levels of IL-6 in the Staph+AgNP/NSP group were similar to the levels of IL-6 in the Staph+AQ and Staph+SS groups. For all three cytokines, AgNP/NSP had a beneficial effect in the excision wound model, when compared with the Staph group (Table 1).

**Table 1:** Cytokine profile of wound healing.

	Untreated	Staph	Staph+NSP	Staph+Poly-Ag	Staph+AgNP/NSP	Staph+AQ	Staph+SS
**Acute burn model**							
VEGF-A	744.0±27.5*	556.2±17.6	517.9±32.4	563.5±40.1	779.8±19.0*	791.4±14.7*	777.7±4.2*
TGF-β1	787.1±19.4*	271.7±22.7	388.9±10.8*	405.1±6.8*	870.2±21.1*	810.6±17.2*	878.8±17.7*
IL-6	148.5±24.1*	547.5±15.8	323.3±10.5*	278.1±12.2*	271.1±14.4*	269.3±11.8*	281.9±11.5*
**Excision model**							
VEGF-A	659.8±26.4*	410.1±16.1	408.5±28.1	575.8±18.7*	650.4±14.8*	667.7±19.8*	599.2±32.2*
TGF-β1	419.7±30.9*	200.1±27.4	225.9±31.7	217.8±41.8	415.8±17.3*	411.8±15.3*	423.0±40.5*
IL-6	482.6±11.4*	799.5±14.2	254.9±20.1*	233.6±17.4*	190.8±24.8*	195.0±13.9*	197.5±13.3*

Serum levels of VEGF-A, TGF-β1 and IL-6 from mice of different treatment groups in both acute thermal injury at day 7 and excision wound at day 15 were examined by ELISA method.

Results are expressed as the mean (pg/ml) ± standard deviation (SD) from three independent experiments of each group.

* Statistically significant as compared to the Staph group (*P*<0.01, Student’s *t* test).

**Figure 3:**
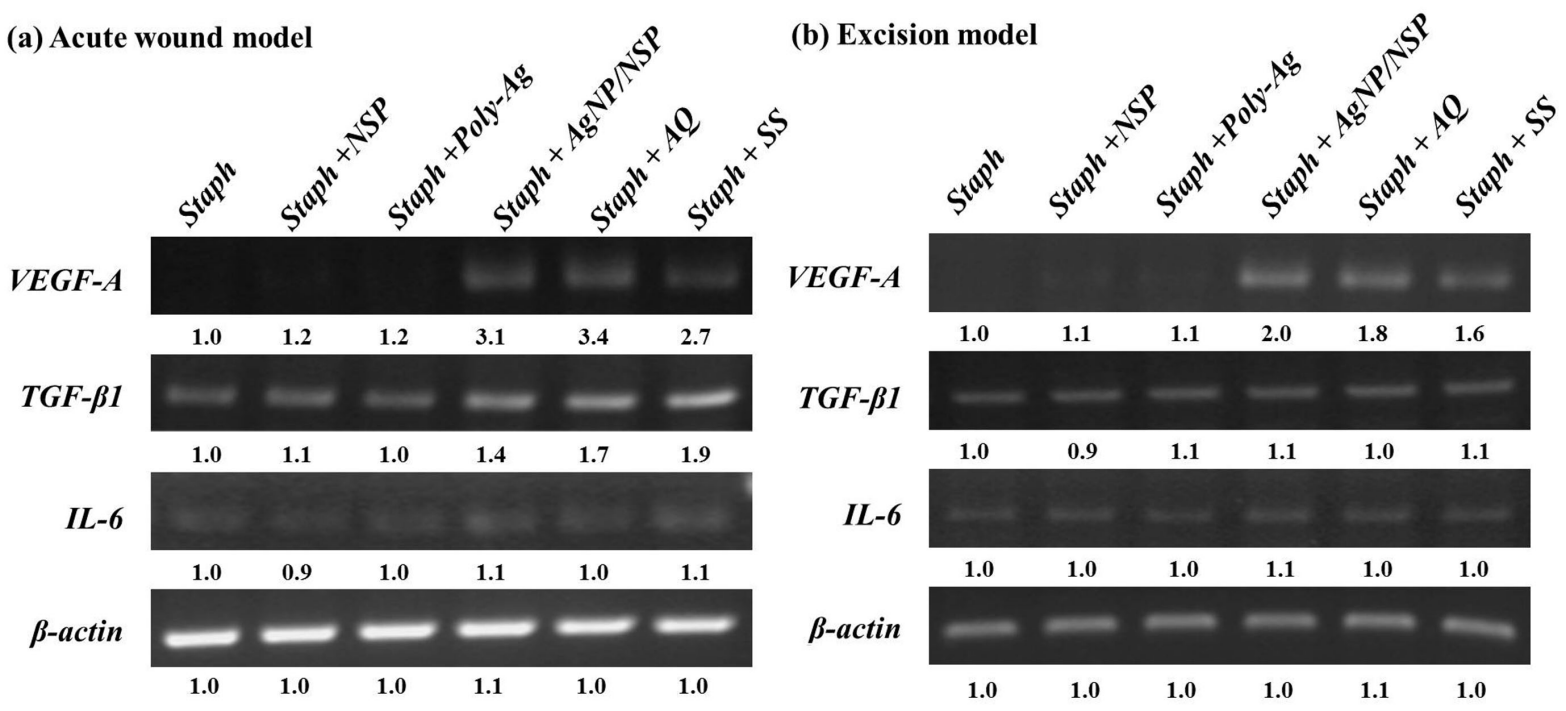
mRNA levels of *VEGF-A, TGF-β1* and *IL-6* in different treatment groups during wound healing. AgNP/NSP treatment also modulates the expression of cytokine mRNAs in wounded skin. The levels of *VEGF-A*, *TGF-β1* and *IL-6* mRNA expression from mice of different treatment groups in both acute thermal injury at day 7 (a) and excision wound at day 15 (b) were examined by RT-PCR. Relative band intensities of different groups were calculated by a densitometer and are demonstrated by the values under the bands. The data shown are representative of three independent experiments.

We have examined the changes in the cytokine levels of the local wound tissue by collecting the wound skin and analyzing the mRNA expression of *VEGF-A*, *TGF-β1*, and *IL-6* at day 7 (acute burn wound) and day 15 (excision wound). In accordance with the circulating levels, in both wound models, the expression of *VEGF-A* mRNA in the Staph+AgNP/NSP, Staph+AQ, and Staph+SS groups were all significantly higher than that in the Staph, Staph+NSP, and Staph+Poly-Ag groups (*P*<0.01). A similar trend was observed for *TGF-β1* mRNA expression when comparing Staph+AgNP/NSP with the other groups in the acute burn wound model. However, in the excision wound model (Fig. 3b), there was no significant difference in the levels of *TGF-β1* mRNA expression. The expression of *IL-6* mRNA in the local wound tissue was similar among the six groups in both models.

In conclusion, the newly developed nanohybrid AgNP/NSP exhibited several functions when applied to the healing of infected mouse skin wounds. The serum levels and local mRNA expression of VEGF-A, TGF-β1, and IL-6 in mice treated with the nanohybrid were similar to those of two conventional silver compounds, AQ and SS. The presence of the NSP supports for the AgNPs resulted in a trend of lower cytotoxicity than for Poly-Ag and SS at 8.75 ppm Ag on fibroblasts and high effectiveness for wound healing. The combination of nanometer-scaled inorganic materials, Ag particles, and silicate platelets represents a novel approach for developing new wound-dressing agents. Further tests are needed to fully understand the molecular mechanisms of these nanomaterials during wound healing.

## Materials and Methods

### Materials

Sodium montmorillonite, a natural smectite aluminosilicate, was obtained from Nanocor Co. (USA). The clay has a generic structure of 2/1 layered silicate/aluminum oxides with 2 tetrahedral sheets sandwiching an edge-shared octahedral sheet and exchangeable Na^+^ counter ions with a cation exchange capacity (CEC) of 120 mequiv/100 g. The NSPs were prepared from the exfoliation of the sodium form of smectite montmorillonite clay by a polyamine salt, followed by a two-phase extraction process as described in previous studies. [16] The NSPs possess a large surface area (720 m^2^/g) and intense surface anionic charges (approximately 18,000 charges/platelet). The copolymer, poly (styrene-co-maleic anhydride) with styrene/maleic anhydride molar ratios of 3/1 in the backbones, was purchased from Aldrich Chemical Co. and designated as SMA3000. The poly (oxyethylene–oxypropylene)-monoamine (POE-amine) with the designated trade name of Jeffamine® M2070 was obtained from Huntsman Chemical Co. The amine is hydrophilic and water-soluble, and it has a backbone of oxyethylene/oxypropylene blocks at 32/10 unit molar ratio with an average molecular weight of 2000. Silver nitrate (AgNO_3_; 99.8%) was purchased from SHOWA Chemical Co.

### Preparation of the AgNP/NSP Nanohybrid

NSPs (0.3 g in 30 g of water; 1.0 wt%) were dispersed in deionized water and mechanically stirred, followed by the addition of AgNO_3_ solution (0.061 g of 1.0 wt% in water) at an equal molar ratio of Ag^+^ to CEC. The process involved the replacement of Ag^+^ with Na^+^ counter ions on the clay surface and the consequent reduction of Ag^+^ by ethanol (18.05 ml). The reaction mixture was stirred vigorously and monitored by observing the color change from yellow to deep-red and was analyzed further with UV-vis spectroscopy and transmission electron microscopy (TEM). It is possible that the natural clay with a layered structure was randomized by ionic exchange exfoliation to afford the thin platelets of NSP. The pristine clay with exchangeable Na^+^ counter ions on the surface was replaced by Ag^+^ and subsequently reduced to stabilized Ag^0^ particles. The stably immobilized AgNPs with an average diameter of 4.9 nm were mostly observed in the platelet boundary. [21] In our earlier studies, the NSP immobilization for AgNPs showed the benefit of preventing Ag from aggregation due to intense van der Waals forces. [25].

### Polymer-Ag (Poly-Ag) Colloidal Solution

The aqueous solution of the polymer (1.5 g) and silver nitrate (0.5 g) was mixed and dissolved in 50 g of deionized water, followed by the addition of NaBH_4_ (0.12 g) in 50 g of distilled water under a nitrogen atmosphere. The solution was stirred at room temperature for 2–3 h. The formation of polymer-dispersed AgNP colloidal solution was monitored with a UV-vis spectrometer and TEM. In this work, we selected a conventional polymeric dispersant for AgNP stabilization and verified the uniqueness of the NSP effect. The exfoliation of layered clay into NSPs for AgNP immobilization, and a comparison of Ag particle size between the NSPs (AgNP/NSP) and the polymeric SMA-M2070 organic dispersant (Poly-Ag) was performed. A similar particle size of around 4–5 nm diameter was observed. [20].

### Cell Culture and Preparation

Primary human foreskin fibroblasts (Hs68) purchased from the American Type Culture Collection (Manassas, VA, USA) were cultured in a complete culture medium composed of Dulbecco’s modified Eagle’s medium (DMEM), 10% fetal bovine serum, 100 U/ml of penicillin, and 100 U/ml of streptomycin. The cells were maintained in a humidified atmosphere of 95% air and 5% CO_2_ at 37°C. Upon reaching confluence, the cells were trypsinized with 0.25% trypsin and re-plated onto 10-cm dishes.

### Cytotoxicity Assay

The viability of the Hs68 cells was determined using the 3-(4,5-dimethylthiazol-2-yl)-2,5-diphenyltetrazolium bromide (MTT) assay (Sigma, USA). [20] The cells were split into 24-well culture plates at a density of 5×10^4^ cells/well in l ml of the culture medium and allowed to attach for 24 h before treatment. After being replaced by media containing AgNP/NSPs, polymer-dispersed AgNPs (Poly-Ag), or silver sulfadiazine (SS) (Sulfasil®; Taiwan Veterans Pharmaceutical Co., Ltd., Taoyuan, Taiwan) at concentrations of 0, 8.75, 17.5, 35, 52.5, and 70 ppm of silver, the cells were incubated at 37°C under 5% CO_2_ for 12 h. Then, MTT solution was added, and the cells were incubated at 37°C for 2 h. The supernatant was removed, and dimethyl sulfoxide (DMSO) was added to each well to dissolve the formazan crystals. Then, the soluble solution from each well was transferred to a 96-well plate. The optical density was measured at 570 nm on an enzyme-linked immunosorbent assay (ELISA) reader. Absorbance of the peak height was correlated through a standard curve to the number of living cells in the culture.

### Comet Assay

The procedures for the comet assay were adopted from the method described by Tice et al. [22] The cells were divided and cultured in 6-cm culture plates at a density of 5×10^5^ cells/plate in 2 ml for 24 h. Then, the Ag/NSP and Poly-Ag solutions at concentrations of 0, 8.75, 17.5, 35, 52.5, and 70 ppm of silver were added. A negative control (with double-distilled water) and a positive control (with 100 µM H_2_O_2_ addition) were performed. After incubation for 24 h at 37°C under 5% CO_2_, the cells were trypsinized. The cell suspensions (1×10^5^ cells/ml; 10 µl) were mixed with 1% low melting agarose (150 µl) at 37°C and rapidly spread onto glass slides coated with 1.5% of normal melting point agarose. The agarose was allowed to solidify by lowering the temperature to 4°C for 20 min. The cells were lysed by immersing the slides in a freshly prepared solution (5 M NaCl, 0.5 M EDTA, 1 M Tris-HCl, 1% Triton X-100, 10% DMSO; pH, 10.0) at 4°C for 40 min. Then the slides were immersed in an alkaline solution (0.3 M NaOH, 1 mM EDTA; pH, >13) at room temperature. After 30 min, the slides were placed in a horizontal gel electrophoresis box containing freshly prepared alkaline electrophoresis buffer (0.3 M NaOH, 1 mM EDTA; pH, >13) at 4°C for 30 min before performing electrophoresis. Electrophoresis was performed at 13 V (1 V/cm) and 300 mA in an ice bath at 4°C for 20 min. Then, the sample slides were rinsed with double-distilled water and immersed in 70% ethanol for 5 min. After the slides were air-dried, 50 µl of diluted propidium iodide (PI) solution was applied to each circle of dried agarose and covered with a cover slip. The samples were immediately examined with a fluorescent microscope. Scores ranging from 0 (undamaged) to 4 (maximally damaged) were estimated according to the tail intensity (size and shape) for 200 randomly selected cells.

### Source and Preparation of Bacteria

The pathogenic strain of *Staphylococcus aureus* BCRC 10451 was purchased from the Bioresource Collection and Research Center at the Food Industry Research and Development Institute (Hsinchu, Taiwan), and cultured in nutrient broth medium at 37°C. The bacteria were grown overnight in nutrient broth (Merck, Darmstadt, Germany), and then, 100 µl of the broth was inoculated into fresh medium to restart the cell cycle. After 3 h of incubation at 37°C, the cells were synchronized at the log phase of the growth curve and grown until they reached an optical density at 600 nm (OD_600_) of 0.5–0.6. Then, 1.5 ml aliquots of the solutions at approximately 1×10^8^ colony forming unit (CFU)/ml were applied on the dressings and placed on the wounds.

### Animal Experiments

Twelve-week-old male BALB/C mice weighing between 20 and 25 g were purchased from the National Health Research Institute (Taipei, Taiwan). The animal use protocol in this study was reviewed and approved by the Institutional Animal Care and Use Committee (IACUC) of the National Taiwan University College of Medicine and College of Public Health. The IACUC approval number is 20080117. All animal work was conducted according to the national and international guidelines. Anesthesia was achieved with Zoletil 50 (20–30 mg/kg; Virbac, France) prior to skin wounding.

The acute burn wound model was performed as described previously, with modification. [6], [19] A burn template was made from a 60-ml plastic syringe by cutting a window (3×2 cm^2^) on the side with the opposite half removed such that a mouse could be held horizontally in the burn template. The dorsum of each mouse was carefully shaved and laid on the burn template after anesthesia. An iron plate of size 1.5×1.5 cm^2^ was roasted at 180°C and placed onto the exposed dorsal skin of each mouse for 20 s. All the mice were then immediately placed in an ice-water bath to stop the burning process. This method was used to form a deep partial-thickness thermal injury. For the excision wound model, the dorsal hair of each mouse was shaved, and a piece of full-thickness skin (1.5×1.5 cm^2^) was excised with sterilized scissors. [6].

In the excision wound and burn models, the mice were divided into 7 groups: (1) untreated control, (2) *Staphylococcus aureus* treatment only (Staph), (3) *Staphylococcus aureus* and NSP treatment (Staph+NSP), (4) *Staphylococcus aureus* and Poly-Ag treatment (Staph+Poly-Ag), (5) *Staphylococcus aureus* and AgNP/NSP treatment (Staph+AgNP/NSP), (6) *Staphylococcus aureus* and Aquacel® treatment (Staph+AQ), (7) *Staphylococcus aureus* and silver sulfadiazine treatment (Staph+SS). For each group, we applied 1.5 ml of *S. aureus* (1×10^8^ CFU/ml) solution to a piece of clean gauze to produce a “bacterial dressing” that was immediately placed on the wounds. The wounds were then covered with the “bacterial dressings” and bandaged for 1 day to let the bacterial solution enter the wound area. The “bacterial dressing” has little disinfectant activity as it only consists of a piece of gauze and contains no medications. One day later, we changed the dressings to the therapeutic dressings corresponding to the different treatments. Each treatment was then topically applied to the wound bed on the second day. The wound dressings were changed daily until sacrifice. While changing the dressings, a close-up picture of each wound was taken, and the wound appearance was inspected by three dermatologists to evaluate the edema, necrosis, exudation, and eschar formation. The mice were sacrificed humanely by decapitation on the 7^th^ day after the injury in the acute burn wound group or on the 15^th^ day for the excision wound group. Tissue samples of the wound were harvested for histological examination and RNA extraction. Blood samples were also obtained and immediately transferred into EDTA-coated tubes. After centrifugation at 3000 *g* for 15 min, the serum samples were removed and stored at −70°C.

### Preparation of Dressings

In both the Poly-Ag and AgNP/NSP groups, the same amount of solution containing 2 mg/ml of each silver preparation was coated on a dressing and applied topically to the wound bed to maintain the silver content at 140 ppm. In the AQ group, a commercialized dressing containing 1.2% silver (140 ppm) in an ionic form (Aquacel®) was applied directly to the wound bed. In the SS group, the mice were treated by the topical application of 1% silver sulfadiazine (95.2 mg) cream (Sulfasil®) on a piece of sterile, non-adherent, absorbent dressing (2×2 cm^2^) to give an equal amount of silver ions released (140 ppm) as the other Ag treatment groups.

### ELISA

Serum levels of vascular endothelial growth factor (VEGF)-A, interleukin (IL)-6, and transforming growth factor (TGF)-β1 were measured with cytokine-specific ELISA kits (Endogen, Woburn, MA, USA). Each measurement was performed according to the manufacturer’s instructions and repeated in triplicate.

### Reverse Transcription Polymerase Chain Reaction

Total RNA extraction from homogenized fresh-skin tissue was performed with commercial kits (BIOTECX Laboratory Inc., Houston, TX, USA). Reverse transcription of the isolated RNA was performed in a final reaction volume of 20 µl containing 5 µg of total RNA in Moloney murine leukemia virus (MMLV) RT buffer (Promega, Madison, WI, USA) consisting of 10 mM dithiothreitol, deoxynucleoside 5′-triphosphates (dNTPs; each at 2.5 mM), 1 µg of (dT) 12–18 primer, and 200 U of MMLV RT. The reaction mixture was incubated at 37°C for 2 h, and the reaction was terminated by heating at 70°C for 10 min. One microliter of the reaction mixture was then amplified by PCR using the following primers:

Mouse VEGF-A, sense 5′-CAGGCTGCTGTAACGATGAA-3′ and antisense 5′-AATGCTTTCTCCGCTCTGAA-3′.

Mouse TGF-β1, sense 5′-TGACGTCACTGGAGTTGTACGG-3′ and antisense 5′-GGTTCATGTCATGGATGGTGC-3′.

Mouse IL-6, sense 5′-GATGCTACCAAACTGGATATAATC-3′ and antisense 5′-GGTCCTTAGCCACTCCTTCTGTG-3′.

Mouse β-actin, sense 5′-AGCCATGTACGTAGCCATCC-3′ and antisense 5′-CTCTCAGCTGTGGTGGTGAA-3′.

The PCR cycling profile consisted 40 cycles of 45 s at 95°C (denaturation), 1 min at 58 or 60°C (annealing), 1 min at 72°C (elongation), and a final 10-min extension at 72°C. The PCR samples were subjected to electrophoresis in 1% agarose gel, which was then stained with ethidium bromide and photographed under UV illumination. Relative band intensities to quantify the mRNA levels for different tests were calculated by a densitometer.

### Statistical Analysis

The two-tailed Student’s *t* test was used for simple comparison of 2 values where appropriate. The data from 3 or 4 independent experiments were expressed as the mean ± SD. A *P*-value of less than 0.05 was considered statistically significant for all the tests. All analyses were performed with the use of SAS software (version 8.02; SAS Institute, Cary, NC, USA).

### Instruments and Analyses

UV-vis spectroscopy of the AgNP solution was performed on a Shimadzu UV-2450 spectrophotometer.
