# Correction: Nanohybrids of Silver Particles Immobilized on Silicate Platelet for Infected Wound Healing

**DOI:** 10.1371/annotation/627cef98-9ea7-4c06-ad0a-f7d2c83a6bc4

**Published:** 2012-10-03

**Authors:** Chia-Yu Chu, Fu-Chuo Peng, Ying-Fang Chiu, Hsing-Chuan Lee, Chien-Wen Chen, Jiun-Chiou Wei, Jiang-Jen Lin

There was an error in the funding statement. The correct funding statement is: The authors acknowledge financial support from National Science Council of Taiwan (NSC 99-2221-E-002-236-MY3), and partially from National Taiwan University under the project of 97R0102. The funders had no role in study design, data collection and analysis, decision to publish, or preparation of the manuscript. 

